# Antioxidant Response during the Kinetics of Anhydrobiosis in Two Eutardigrade Species

**DOI:** 10.3390/life12060817

**Published:** 2022-05-30

**Authors:** Ilaria Giovannini, Paola Antonia Corsetto, Tiziana Altiero, Gigliola Montorfano, Roberto Guidetti, Angela Maria Rizzo, Lorena Rebecchi

**Affiliations:** 1 Department of Life Sciences, University of Modena and Reggio Emilia, 41125 Modena, Italy; ilaria.giovannini@unimore.it (I.G.); roberto.guidetti@unimore.it (R.G.); 2 Department of Pharmacological and Biomolecular Sciences, University of Milan, 20122 Milan, Italy; paola.corsetto@unimi.it (P.A.C.); gigliola.montorfano@unimi.it (G.M.); 3 Department of Education and Humanities, University of Modena and Reggio Emilia, 42121 Reggio Emilia, Italy; tiziana.altiero@unimore.it

**Keywords:** anhydrobiosis, desiccation stress, ROS, scavenging enzymes, tardigrades

## Abstract

Anhydrobiosis, a peculiar adaptive strategy existing in nature, is a reversible capability of organisms to tolerate a severe loss of their body water when their surrounding habitat is drying out. In the anhydrobiotic state, an organism lacks all dynamic features of living beings since an ongoing metabolism is absent. The depletion of water in the anhydrobiotic state increases the ionic concentration and the production of reactive oxygen species (ROS). An imbalance between the increased production of ROS and the limited action of antioxidant defences is a source of biomolecular damage and can lead to oxidative stress. The deleterious effects of oxidative stress were demonstrated in anhydrobiotic unicellular and multicellular organisms, which counteract the effects using efficient antioxidant machinery, mainly represented by ROS scavenger enzymes. To gain insights into the dynamics of antioxidant patterns during the kinetics of the anhydrobiosis of two tardigrade species, *Paramacrobiotus spatialis* and *Acutuncus antarcticus*, we investigated the activity of enzymatic antioxidants (catalase, superoxide dismutase, glutathione peroxidase, and glutathione reductase) and the amount of non-enzymatic antioxidants (glutathione) in the course of rehydration. In *P. spatialis*, the activity of catalase increases during dehydration and decreases during rehydration, whereas in *A. antarcticus*, the activity of superoxide dismutase decreases during desiccation and increases during rehydration. Genomic varieties, different habitats and geographical regions, different diets, and diverse evolutionary lineages may have led to the specialization of antioxidant strategies in the two species.

## 1. Introduction

Global climate change and its subsequent impacts, such as environmental drought, could be responsible for the decrease or disappearance of several wild species [1]. Under these adverse conditions, desiccation tolerance is a valuable mechanism for escaping the hostile consequences of progressive environmental desiccation. Among the different degrees of desiccation tolerance within taxa, anhydrobiosis is the reversible capability of an organism to tolerate a severe loss of its body water by evaporation following the progressive desiccation of its surrounding habitat [2]. Anhydrobiosis is one of the most peculiar adaptive strategies existing in nature since, due to the absence of an ongoing metabolism, an organism in the anhydrobiotic (desiccated) state lacks all the dynamic features (e.g., movement, growth, feeding, reproduction, etc.) of living beings. In that sense, it is not alive, but still not dead since rehydration reactivates the organism’s metabolism [3,4]. Anhydrobiosis has originated independently several times in the history of life, being present in diverse evolutionary lines within groups of unicellular and multicellular organisms [3,5,6].

Even though anhydrobiosis has a great adaptive potential, in animals, it is found in only a restricted number of invertebrate taxa whose body sizes do not exceed 5–7 mm in length but are often much smaller [5,7]. This apparent morphological limit is linked to the capability to tolerate physical and physiological constraints [5]. Moreover, terrestrial and phytopathogenic nematodes, bdelloid rotifers, and limno-terrestrial tardigrades are able to enter anhydrobiosis at any stage of their life cycle [8], while in other animals (e.g., small crustaceans and the African midge), anhydrobiosis is restricted to eggs or juvenile stages [9]. Abiotic factors, such as high temperatures, a high relative humidity, a high oxygen partial pressure, and time spent in a desiccated state can reduce anhydrobiotic survival, which ultimately results in the death of organisms as a consequence of membrane destabilization, protein and nucleic acid denaturation, metabolic dysregulation, and oxidative stress [8,10,11].

Oxidative stress seems to be one of the most deleterious consequences related to anhydrobiosis because water depletion increases the ionic concentration with the production of high amounts of reactive oxygen species (ROS) [12,13]. These effects were demonstrated in several anhydrobiotic organisms, such as cyanobacteria [14], yeast [15], seaweeds [16], mosses [17], shrimps [18], and tardigrades [19,20]. ROS are not always enemies. Indeed, they are generated in most cell compartments during the normal vital processes of cells (e.g., the electron transport processes in photosynthesis and cell respiration, enzyme-catalysed reactions) [21,22,23], serve as second messengers, and regulate several physiological functions [24]. Nevertheless, an imbalance between the increased production of ROS and the limited action of antioxidant defences is a source of damage, such as the denaturation of proteins, the peroxidation of lipids, and alterations of nucleic acids [10,25,26,27,28,29]. In addition, ROS are involved in the development of many important chronic human diseases and play a major role in the ageing process [28,30]. Organisms use antioxidant defence systems to obtain protection against ROS. These defence systems are distributed in all cell compartments and are represented by enzymatic or non-enzymatic antioxidants that intercept ROS before they damage molecules [12,29,31,32,33,34,35,36].

**Figure 1:**
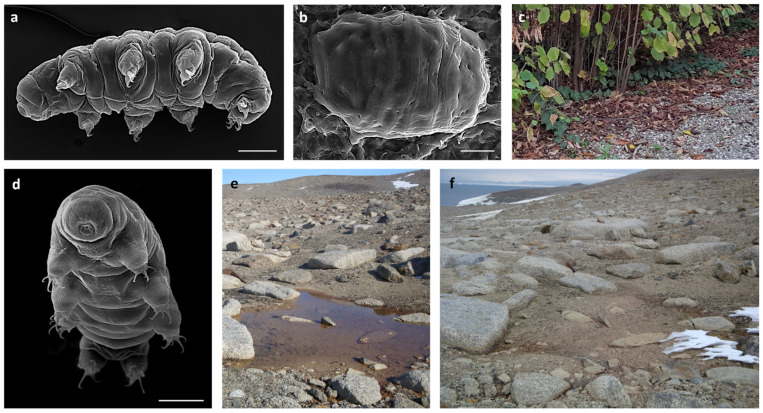
(**a**) *Paramacrobiotus spatialis in toto* (SEM). (**b**) Desiccated specimen of *P. spatialis* (SEM). (**c**) Hazel-leaf litter inhabited by *P. spatialis* (Formigine, Modena, Italy). (**d**) *Acutuncus antarcticus in toto* (SEM). (**e**) Temporary freshwater pond at Victoria Land (Antarctica) inhabited by *A. antarcticus*. (**f**) The same pond in (**e**) in a desiccated state. Scale bars: 30 µm.

Anhydrobionts seem to exhibit a control of ROS production during the kinetics of anhydrobiosis using an efficient antioxidant machinery mainly represented by ROS scavenger enzymes, as evidenced by comparing the activities of scavenger enzymes in hydrated and desiccated organisms [34,35,37,38]. Antioxidants could also be involved during rehydration, but available data revealed that they could increase, decrease, or be unaffected depending on the taxa and on the desiccation/rehydration processes [35,36,39,40]. To gain insights into the dynamics of antioxidant patterns during the kinetics of anhydrobiosis, we investigated the activity of enzymatic antioxidants and the amount of non-enzymatic antioxidants in the course of rehydration after a period of experimental desiccation in two tardigrade species [*Paramacrobiotus spatialis* Guidetti et al., 2019 and *Acutuncus antarcticus* (Richters, 1904); Figure 1]. These two species were selected based on their high desiccation tolerance, even though they inhabit diverse environments and belong to two different evolutionary lines [41,42,43,44]. *Paramacrobiotus spatialis* was previously used as a model system to characterize enzymatic (catalase, superoxide dismutase, glutathione peroxidase, and glutathione reductase) and non-enzymatic (glutathione) antioxidants, although only two static and opposite physiological conditions of the desiccation process were considered: hydrated (active) and anhydrobiotic (desiccated) states [37]. By contrast, no previous antioxidant studies have been carried out on *A. antarcticus* or on any Antarctic tardigrades.

## 2. Material and Methods

### 2.1. Model Species 

For the experiments, two model species were used: *Paramacrobiotus spatialis* and *Acutuncus antarcticus*.

*Paramacrobiotus spatialis* (Eutardigrada; Macrobiotidae) colonises leaf litter. It is an omnivorous species able to feed on fluids of vegetal cells and algae and to prey on rotifers, nematodes, and other tardigrade species [45]. The species is bisexual and amphimictic [44]. It has an exceptional anhydrobiotic capability [41] and was previously used to investigate the molecules involved in desiccation tolerance [20,37,46]. In addition, it has been used as a model species to investigate the ability to tolerate ultraviolet radiation, freezing, and stresses associated with the environment of space [41,47,48,49,50,51]. Specimens used in this study came from hazel-leaf litter collected in Northern Italy (Formigine, Modena, Italy; 44°34.253′ N, 10°50.892′ E, 80 m a.s.l.; Figure 1c). 

Leaf litter containing tardigrades was naturally desiccated in the laboratory and kept at −80 °C under vacuum until its use. To extract tardigrades from leaf litter, the sample was moisturised by sprinkling tap water on it, allowing a gradual hydration of the animals for at least 10 min. Then it was completely immersed in tap water for about 30 min and sieved with two sieves (meshes: 500 µm and 38 µm). Animals were picked up from the sieved material with a glass pipette under a stereomicroscope and starved in rearing water (mineral natural water/distilled water, 1:1) in controlled conditions (14 °C for 12 h of dark/12 h of light) for 24 h before their use.

*Acutuncus antarcticus* (Eutardigrada; Hypsibiidae) is a pan-Antarctic species and represents the most widespread and common Antarctic tardigrade [43]. It is an herbivorous/bacteriophagous species very abundant in temporary freshwater ponds [43,52] and is parthenogenetic [52]. In addition, it has been used as a model species to investigate the ability to tolerate desiccation, freezing, ultraviolet radiation, and increasing temperatures due to global warming [42,53]. The specimens used in this study came from a laboratory culture started from specimens collected in a temporary freshwater pond in Antarctica (Victoria Land, Terranova Bay, 74°42.580′ S, 164°06.086′ E, 125 m a.s.l.; Figure 1e,f). Cultured animals were kept in water at 14 °C with a photoperiod of 12 h/12 h (L/D) and *Chlorococcum* sp. as a food source (for the detailed culturing protocol, see Altiero et al. [52]).

Animals were picked up from the culture with a glass pipette under a stereomicroscope and starved in rearing water (mineral natural water/distilled water, 1:1) in controlled conditions (14 °C for 12 h/12 h L/D) for 24 h before their use.

### 2.2. Dehydration and Rehydration Processes

#### 2.2.1. Desiccation Protocol

Animals of both species were dehydrated in the laboratory under controlled conditions for air relative humidity (RH) and temperature using a climate chamber (CHL, Angelantoni Industrie, Milan, Italy). A group of animals (see *Experimental Groups* below) was placed on a small Whatman filter paper (square of 1 cm^2^) with a drop of rearing water. The paper was introduced in the climate chamber and exposed to 80% RH for 4 h, then exposed to 50% RH for 4 h, and finally, left at 0–3% RH at room temperature overnight [20,41,48,54]. To desiccate specimens of *A. antarcticus*, the temperature of the climate chamber was set at 14 °C [42], while for *P. spatialis*, it was set at 18 °C [20,41,48]. The protocol of desiccation is standardized and allows animals to reach high survival rates after rehydration [20,41,42,48,54].

#### 2.2.2. Rehydration Protocol

In order to rehydrate animals, 8 µL of rearing water was slowly added to each filter paper every 10 min for a total of 60 min. Then, animals were immersed in rearing water for 1 h (t_1_) or for 24 h (t_24_) at 14 °C according to the experimental groups. The protocol of rehydration is standardized and allows animals to reach high survival rates, as evidenced by the recovery of animal locomotion performance [20,41,42,48,54].

### 2.3. Experimental Groups

For both species, animals in different phases of the anhydrobiotic process were used: (A) desiccated, (B) rehydrated for 1 h, (C) rehydrated for 24 h, and (D) hydrated. These experimental groups were as follows.

(A) Desiccated (dry): Animals were dehydrated using the protocol described above and kept in a desiccated state for 1 day. Then, they were removed from the filter paper using tweezers, frozen in liquid nitrogen within a plastic tube to facilitate the breakage of the animal cuticle, and finally, used for enzyme extraction (see below). 

(B) Rehydrated for 1 h (t_1_): Animals were dehydrated using the protocol described above, kept desiccated for 1 day, rehydrated using the protocol described above, kept in rearing water for 1 h, and frozen in liquid nitrogen within a plastic tube before enzyme extraction (see below).

(C) Rehydrated for 24 h (t_24_): Animals were dehydrated using the protocol described above, kept desiccated for 1 day, rehydrated using the protocol described above, kept in water for 24 h, and frozen in liquid nitrogen within a plastic tube before enzyme extraction (see below).

(D) Hydrated (Ctr): Animals were extracted from the substrate, starved, kept in rearing water for 24 h, and frozen in liquid nitrogen within a plastic tube before enzyme extraction (see below). They represented the control group.

For group (A), 3 replicates each made up of 100 animals were used. For groups B, C, and D, 3 replicates each made up of 50 animals were used.

### 2.4. Biochemical Assay

Each group of frozen tardigrades was homogenised with a potter on ice using 3 cycles of 30 s for each group. The homogenate was assayed for protein content according to Lowry et al. [55] and then used for biochemical assay after checking animal disaggregation under stereomicroscope, as reported by Rizzo et al. [37]. The following scavenging enzymes were tested: superoxide dismutase (SOD; EC: 1.15.1.1), catalase (CAT; EC: 1.11.1.6), glutathione reductase (GR; EC: 1.8.1.7), and glutathione peroxidase (GPx; EC: 1.11.1.9). The total glutathione (GSH) content was also determined. Substrates and reagents for biochemical determinations were: NAD(P)H, DTNB (5,5′- dithio-bis-2-nitrobenzoic acid), GSH, GSSG, glutathione reductase, and *tert*-butyl hydroperoxide [37,41]. For each tested molecule, homogenates were analysed in triplicates.

#### 2.4.1. Enzyme Activity Assays

The enzyme activity assays were carried out as reported by Rizzo et al. [37]. In particular, the enzyme activity of SOD was assayed using the method based on NAD(P)H oxidation inhibition according to Paoletti and Mocali [56]. The enzyme activity of CAT was evaluated by measuring the consumption of H_2_O_2_ according to Aebi [57]. The enzyme activity of GR was assayed following the oxidation of NADPH according to Pinto et al. [58]. The enzyme activity of selenium-dependent GPx was assayed according to Prohaska and Ganther [59] following a decrease in the absorbance at 340 nm for 3 min, which corresponds to the rate of GSH oxidation to GSSG in the presence of NADPH and glutathione reductase. 

#### 2.4.2. Determination of the Total Glutathione (GSH)

Each group of tardigrades was homogenised on ice in 5% metaphosphoric acid; the homogenate was centrifuged at 5000× *g* for 10 min at 4 °C, and the homogenate was assayed according to Griffith [60] with some slight modifications, as reported by Rizzo et al. [37].

### 2.5. Statistical Analysis

Differences in the activity of enzymes and in the content of GSH among experimental groups were evaluated through analysis of variance (ANOVA) with the *t*-test. The software programme SPSS 20 (SPSS Inc., Chicago, IL, USA) was used.

## 3. Results

In both tardigrade species, the measurements of ROS-scavenging enzyme activities (superoxide dismutase, catalase, glutathione reductase, and glutathione peroxidase) and the contents of glutathione were taken in relation to mU/µg protein and n moles/µg proteins, respectively. 

### 3.1. Paramacrobiotus spatialis

**Figure 2:**
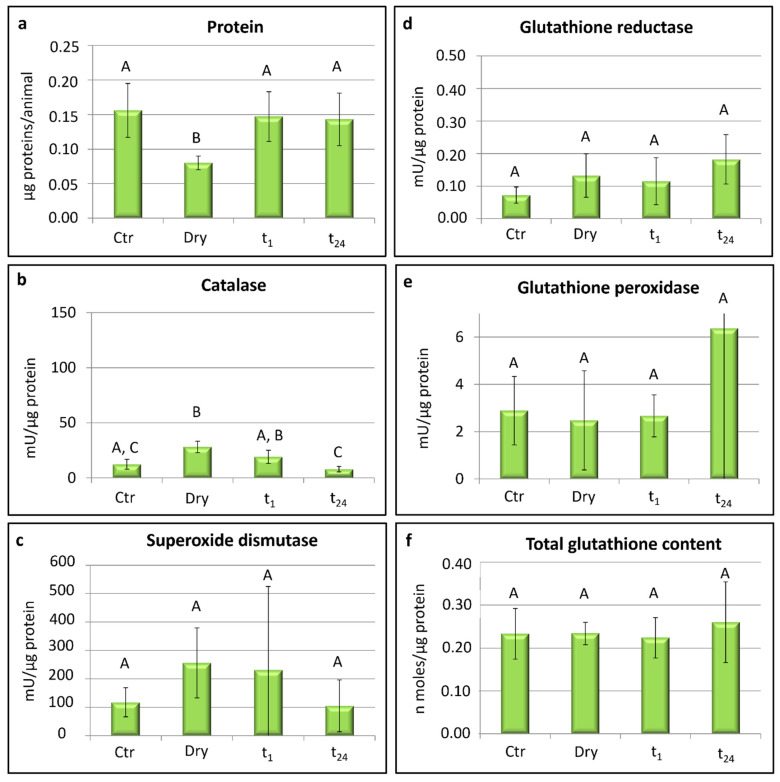
*Paramacrobiotus spatialis*: protein content, activity of scavenger enzymes, and glutathione contents. Ctr = hydrated tardigrades used as controls; dry = desiccated specimens; t_1_ = specimens desiccated then kept in water for 1 h after rehydration; t_24_ = specimens desiccated then kept in water for 24 h after rehydration. Each column represents the mean value of three replicates. The bar on each column represents the standard deviation. Different letters above the columns indicate significant differences between groups, whereas shared letters indicate no significant differences.

In *P. spatialis*, the content of total proteins detected in the desiccated specimens (dry) was significantly lower with respect to that of the proteins measured in the other groups: the hydrated animals (Ctr; *t*-test: *t* = 3.30; *p* < 0.05), tardigrades rehydrated for 1 h (t_1_; *t*-test: *t* = −3.12; *p* < 0.05), and animals rehydrated for 24 h (t_24_; *t*-test: *t* = −2.79; *p* ≤ 0.05; Figure 2a). 

Significant differences were recorded in the activity of catalase among the experimental groups (one-way ANOVA: F_(3,8)_ = 10.31; *p* < 0.01; Figure 2b). In particular, the highest activity of catalase was recorded in the desiccated specimens (dry) with respect to controls (Ctr; *t*-test: *t* = −3.97; *p* < 0.05) and the rehydrated animals for 24 h (t_24_; *t*-test: *t* = 5.98; *p* < 0.01). Further differences were detected in the specimens rehydrated for 1 h (t_1_) with respect to those rehydrated for 24 h (t_24_; *t*-test: *t* = 2.99; *p* < 0.05; Figure 2b). No significant differences were recorded in the catalase activity between the controls and animals rehydrated for 24 h (t_24_) when the activity was the lowest. Moreover, no significant differences were detected among the four experimental groups in the activity of superoxide dismutase, glutathione reductase, and glutathione peroxidase or in the glutathione contents (Figure 2c–f).

### 3.2. Acutuncus antarcticus

**Figure 3:**
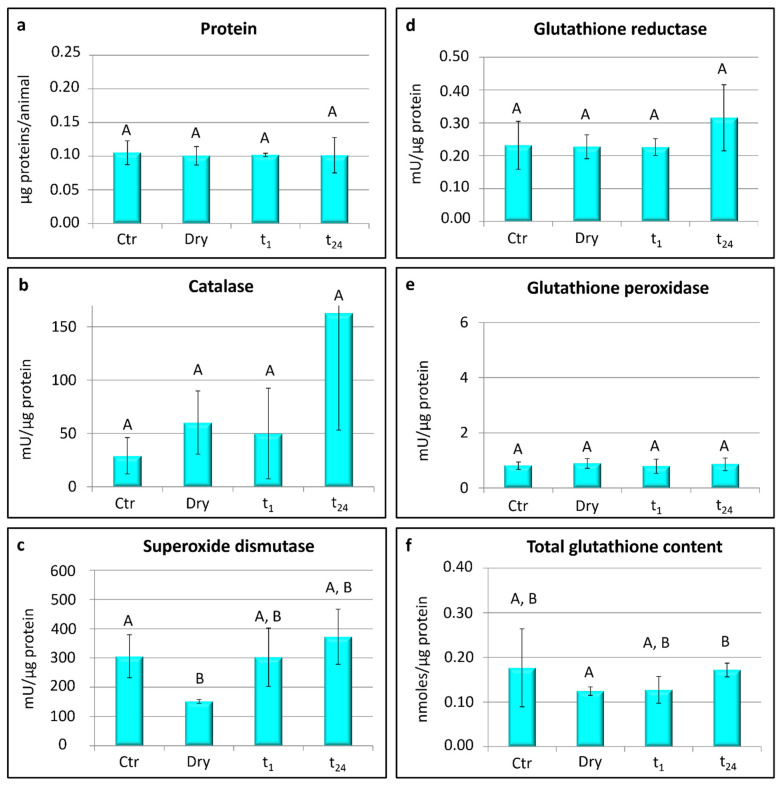
*Acutuncus antarcticus*: protein content, activity of scavenger enzymes, and glutathione content. Ctr = hydrated tardigrades used as controls; dry = desiccated specimens; t_1_ = specimens desiccated then kept in water for 1 h after rehydration; t_24_ = specimens desiccated then kept in water for 24 h after rehydration. Each column represents the mean value of three replicates. The bar on each column represents the standard deviation. Different letters above the columns indicate significant differences between groups, whereas shared letters indicate no significant differences.

In *A. antarcticus*, no significant differences were detected in the protein contents between the four experimental groups (Figure 3a). 

Significant differences were found in the activity of superoxide dismutase among the groups (one-way ANOVA: F_(3,8)_ = 4.33; *p* < 0.05; Figure 3c). In particular, the activity of superoxide dismutase was lower in the desiccated animals (dry) with respect to controls (Ctr; *t*-test: *t* = 3.62; *p* < 0.05; Figure 3c), whereas no significant differences were detected in its activity between the control and rehydrated animals (t_1_ and t_24_; Figure 3c). No significant differences were detected among the experimental groups in the activity of glutathione reductase, glutathione peroxidase, and catalase (Figure 3b,d,e). The total content of glutathione was significantly lower in the desiccated specimens (dry) than in the specimens rehydrated for 24 h (t_24_; *t*-test: *t* = −4.51; *p* < 0.05; Figure 3f), whereas no significant differences were detected between the other experimental groups.

## 4. Discussion

Since the discovery of anhydrobiosis, a huge amount of data covering different and complementary topics has been published with the aim of understanding the secret of life without water. It is known that anhydrobionts evolved to have morphological, physiological, biochemical, and molecular adaptations to withstand the drastic loss of intracellular water (e.g., [7,61,62,63,64,65,66]), but to date, what kind of biochemical and molecular patterns permit the preservation of cells and, consequently, such an organism’s viability is still an open question. This study contributed to this question by investigating the activity of enzymatic antioxidants and the amount of non-enzymatic molecules in counteracting reactive oxygen species (ROS) produced in the course of rehydration [14,15,16,17,18,19,20]. A biological antioxidant is a compound able to delay or prevent the oxidation of a substrate. Among their functions are lowering oxidative stress, DNA mutations, and malignant transformations, as well as lowering other parameters of cell damage [67]. Antioxidants are classified as enzymatic (e.g., superoxide dismutase, catalase, glutathione peroxidase, and glutathione reductase), non-enzymatic (e.g., glutathione and proteins such as ferritin, transferrin, ceruloplasmin, and even albumin) and low molecular-weight scavengers (e.g., uric acid, coenzyme Q, and lipoic acid) [68]. However, the induction of protective mechanisms, such as the antioxidant system, in response to desiccation requires a minimal time to be stimulated and activated [36]. 

The choice of tardigrades as model organisms to study biochemical pathways of desiccation tolerance is linked to their ability to perform anhydrobiosis in order to colonise and persist in stochastic habitats subject to repeated desiccation and rehydration events (Figure 1c,e,f), even though they are aquatic animals and require at least a film of water surrounding their bodies to exhibit active life [8]. In tardigrades, anhydrobiosis causes a significant increase of both ROS-damaged proteins, accumulated as carbonylated products [19], and ROS production occurring during the desiccation process and in the desiccated state [20]. Tardigrades have the capability to mitigate oxidative stress thanks to a specialization in the antioxidative enzyme system inferred by the presence of multiple gene repertoire traits in the tardigrade genome that allow them to have an enhanced tolerability [69].

It is well known that the first line of defence against ROS toxicity involves the system composed of catalase (CAT) and superoxide dismutase (SOD) enzymes in which the superoxide dismutase depletes the superoxide radical anion (O_2_·^−^), converting it into H_2_O_2_, while the catalase decomposes H_2_O_2_ in water and oxygen [67,70]. Among the antioxidant molecules tested in the tardigrade *P. spatialis* specimens, only the catalase activity changed significantly during the entire cycle of the dehydration/rehydration process. In particular, the activity of the CAT enzyme increased during the dehydration process, reaching the highest value in desiccated animals, and decreased again during the rehydration, coming back to the values of the control animals after 24 h in water (Figure 2b). This indicates the gene expression of catalase increases during dehydration and decreases during rehydration in order to counteract ROS produced under desiccation. This is in line with a recent study on RNA interference in *P. spatialis* [20], evidencing the important role of catalase during the rehydration phase, when the return of water allows for the restarting of biochemical reactions. Giovannini et al. [20] targeted the tardigrade catalase gene to disrupt its function, and they found that targeted animals were immobile at an initial phase of rehydration but were able to recover motility in the course of a rehydration. The targeted specimens were not able to produce the catalase enzyme during desiccation and thus, they did not have enough catalase to act during rehydration, leading to an immobilization of the tardigrades at the beginning of the rehydration [20].

In addition, a similar up-regulation of the catalase gene during the desiccation process and a successive decrease of its expression during rehydration were detected in the larvae of the desiccation tolerant insect *Polypedilum vanderplanki* [29,71,72], indicating that catalase and antioxidants—synthetized before entering the dry state—neutralize, at the early stage of rehydration, the ROS produced at the onset of desiccation. Other evidence of an increase of catalase activity after desiccation comes from several other organisms: the tardigrade *Hypsibius exemplaris* (see Yoshida et al. [73]), the nematodes *Aphelenchoides fragariae* (see Fu et al. [74]) and *Caenorhabditis elegans* (see Erkut et al. [75]), the midge *Belgica antarctica* (see Lopez-Martines et al. [76]), the black tiger shrimp *Penaeus monodon* (see Duan et al. [18]), the lichen microalgae *Trebouxia* sp. (see Hell et al. [40]), and the moss *Sanionia uncinata* (see Pizarro et al. [34]). In this study, although no significant differences in the activity of the catalase enzyme among the experimental groups were evidenced in the tardigrade *A. antarcticus* (Figure 3b), the catalase activity appeared to be higher in the desiccated animals. Moreover, the activity of superoxide dismutase in this species showed significant differences, and it reached the lowest value in desiccated animals with respect to the hydrated (control) and rehydrated animals (Figure 3c). A similar pattern for SOD enzyme activity was also evidenced in the Antarctic moss *S. uncinata*, in which the antioxidant activity of SOD was negatively affected by desiccation, decreasing its activity by about 50% [34]. Moreover, the SOD activity decreased during desiccation in the lichens *Ramalina lacera* [39] and *Asterochloris erici* [36]. The reduction of the SOD activity in dried organisms may be related to a lower gene expression or decreased activity after a first phase of dehydration. It can be supposed that SOD acts at the beginning of the desiccation process, converting the superoxide radical in a less reactive molecule, and then hydrogen peroxide is detoxified into water by the action of CAT. 

Recent genomic, transcriptomic, and proteomic studies showed that the catalase gene family seems expanded within eutardigrades (four copies of the catalase gene), while the picture of the catalase gene family is more complex in heterotardigrades [65]. Regarding SOD, different gene copies ranging from 8 to 17 were revealed in tardigrades [65]. Therefore, genomic varieties and different antioxidant gene expressions that have emerged among species may justify the different antioxidant responses after the dehydration process between *P. spatialis* and *A. antarcticus*. 

Among the non-enzymatic antioxidants, the water-soluble antioxidant glutathione is the most abundant and ubiquitous low molecular-weight thiol in cells [35]. It plays a crucial role in antioxidant defences against ROS and the detoxification of xenobiotics and toxic agents [77,78]. In addition, it protects water-soluble proteins [67]. In *A. antarcticus*, the content of glutathione varied significantly between the desiccated tardigrades and those rehydrated for 24 h (Figure 3f). It reached the lowest amount in the desiccated animals and increased again in the rehydrated animals. Therefore, glutathione is produced ex novo during rehydration. Low levels of glutathione were also recorded in different stages of the larvae of the amphibian *Bombina variegata* developing under decreasing water availability and exhibiting increased oxidative damage [79]. Conversely, the content of glutathione did not change among experimental conditions in *P. spatialis* (Figure 2f), evidencing the induction of different antioxidant responses in the two tardigrade species. *Paramacrobiotus spatialis* relies on the GSH-dependent enzymes (e.g., glutathione peroxidase and glutathione reductase) to scavenge free radicals during the desiccation and rehydration process [20]. The enzyme glutathione peroxidase (GPX) is widely distributed in cells, and it has a high degree of affinity for H_2_O_2_, even though it reduces free hydrogen peroxide when its concentration is very low [18,80]. The enzyme glutathione reductase (GR) reduces the glutathione disulphide to glutathione [12,32]. In this study, no differences in either tardigrade species were found in the activity of GPX and GR enzymes (Figure 2d,e and Figure 3d,e). Moreover, the activity was always detected in the hydrated and dried specimens, and the activity of GPX reached higher values than GR. These high levels of GPX activity reflect the crucial role played by this enzyme during every phase of anhydrobiosis in *P. spatialis* since animals targeted for GPX gene disruption by RNAi are not able to survive after a cycle of desiccation and rehydration [20]. Furthermore, a Mn-dependent peroxidase has been identified in tardigrades, and it contributes to tardigrade anhydrobiosis, as evidenced by a 2.5-fold induction of its gene expression during the slow desiccation of *Ramazzottius varieornatus* [81].

Lastly, with regards to the lowest content of proteins detected in the desiccated specimens of *P. spatialis* (Figure 2a), we can suppose that during the desiccation process, some proteins form oligomers and eventually undergo a sol–gel transition in tardigrade cytosol. In this form, the proteins were not detectable from our assay. Another possible explanation is related to their use as an energy source during the desiccation process.

In this study, distinct mechanisms involved in the kinetics of anhydrobiosis and antioxidant responses were evidenced in the two studied tardigrade species (*A. antarcticus* and *P. spatialis*). Genomic varieties, different antioxidant gene expressions, different habitats (freshwater and terrestrial), different geographical regions (polar and temperate), different diets (herbivorous/bacteriophagous and omnivorous), and diverse evolutionary lineages (Hypsibiidae and Macrobiotidae) may have led to the specialization of antioxidant strategies to counteract oxidative stress.
